# High genetic diversity of ancient horses from the Ukok Plateau

**DOI:** 10.1371/journal.pone.0241997

**Published:** 2020-11-12

**Authors:** Nadezhda V. Vorobieva, Alexey I. Makunin, Anna S. Druzhkova, Mariya A. Kusliy, Vladimir A. Trifonov, Kseniya O. Popova, Natalia V. Polosmak, Vyacheslav I. Molodin, Sergei K. Vasiliev, Michael V. Shunkov, Alexander S. Graphodatsky

**Affiliations:** 1 Department of the Diversity and Evolution of Genomes, Institute of Molecular and Cellular Biology SB RAS, Novosibirsk, Novosibirsk Oblast, Russia; 2 Paleogenomics Laboratory, Novosibirsk State University, Novosibirsk, Novosibirsk Oblast, Russia; 3 Paleometal Archeology Department, Institute of Archaeology and Ethnography SB RAS, Novosibirsk, Novosibirsk Oblast, Russia; University of Florence, ITALY

## Abstract

A growing number of researchers studying horse domestication come to a conclusion that this process happened in multiple locations and involved multiple wild maternal lines. The most promising approach to address this problem involves mitochondrial haplotype comparison of wild and domestic horses from various locations coupled with studies of possible migration routes of the ancient shepherds. Here, we sequenced complete mitochondrial genomes of six horses from burials of the Ukok plateau (Russia, Altai Mountains) dated from 2.7 to 1.4 thousand years before present and a single late Pleistocene wild horse from the neighboring region (Denisova cave). Sequencing data indicates that the wild horse belongs to an extinct pre-domestication lineage. Integration of the domestic horse data with known Eurasian haplotypes of a similar age revealed two distinct groups: the first one widely distributed in Europe and presumably imported to Altai, and the second one specific for Altai Mountains and surrounding area.

## Introduction

The horse domestication process started about 5 thousand years ago and involved severe population bottlenecks, similar to other animals [1]. One of the key features of this process was a drastic difference between female (mare) and male (stallion) trajectories. Mares were supposedly consistently restocked from the surrounding wild populations, which manifested in unprecedented retention of the vast part of mitochondrial variation in the domestic breeds [1, 2, 3–11]. In contrast, stallion variation was limited to a single Y-chromosomal haplotype retained in most modern horses [12], although higher diversity existed in the Iron Age [1, 7, 10, 11].

It is clear that in order to get insights into the details of early domestication one should compare haplotypes of both wild and domestic animals from different locations and to investigate the transportation routes between these sites in ancient times. A comprehensive study involving hundreds of ancient and modern horses [6] demonstrated that the analysis of 247 bp fragment of mitochondrial DNA control region is sufficient for horse genotyping. However, a more detailed analysis of the whole control region has demonstrated the presence of recurrent mutations, affecting the topology of the phylogenetic tree. Therefore, complete mitochondrial DNA analysis seems to be a method of choice for improving the phylogeny robustness [8]. Incorporation of ancient horse samples helps to provide temporal dimension to this type of analysis [9, 13]. Unfortunately, for a large set of recently published ancient horse genomes [1, 10, 11] the underlying mitochondrial genome sequences have not been made publicly available.

**Fig 1:**
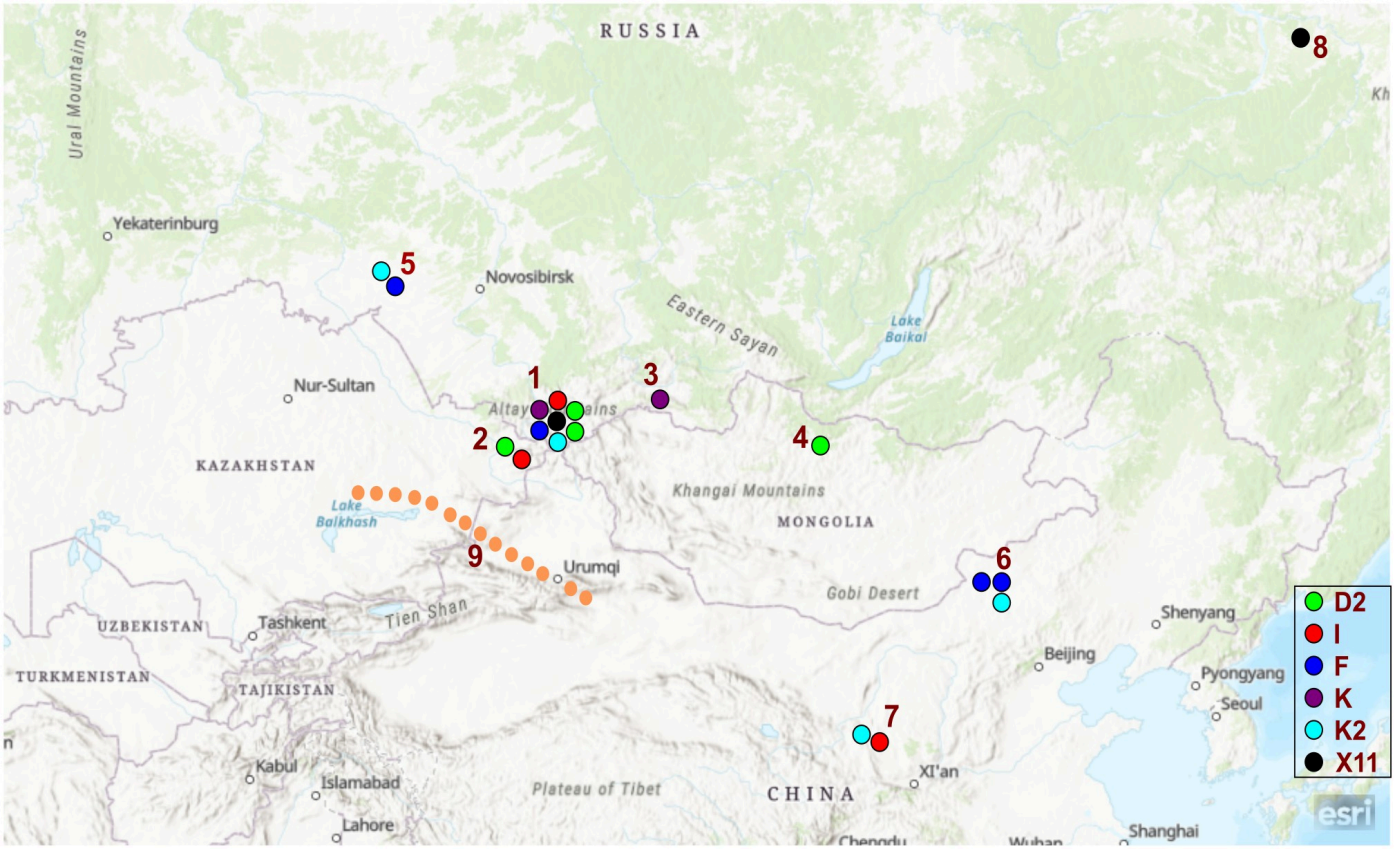
The geographical location of samples. (1) The Ukok Plateau (studied samples) (South Siberia, Russia), (2) The Berel site (North-East Kazakhstan), (3) Tuva (South Siberia, Russia), (4) Mongolia, (5) the Tartas site (West Siberia, Russia), (6) Banchneng and Xiaoshuanggucheng sites (China), (7) the Yujiazhuang site (China), (8) Yana Basin (North-East Siberia, Russia), (9) the northern branch of the Great Silk Road. Samples 2–8 are from [6]. The figure was made based on the map in the USGS National Map Viewer (public domain) resource, it is similar but not identical to the original image and is therefore for illustrative purposes only.

Here we studied DNA from ancient horses found in Iron Age burials of the Ukok Plateau. The Ukok Plateau is located in the South of the Altai Republic (Russia), close to the borders of Kazakhstan, Mongolia, and China at an elevation of 2200–2500 m above the sea level (Fig 1). The plateau became famous after excavations of well-preserved Scythian burials attributed to the Pazyryk culture characteristic to Altai of the early Iron Age [14]. The unique artefacts made of organic materials and two well preserved mummies were discovered due to the location of some burials in permafrost [15]. Based on the records of 2003, remains of horses were found in 212 of 569 excavated kurgans of the Pazyryk culture (135 burials) [16], but their genetic diversity has not been systematically studied yet. Here we studied the horse bones from burials of two major ancient archaeological sites located 20 km from each other: Ak-Alakha and Verkh-Kaldzhin. Six bone samples were collected in different burials to exclude the possibility of analyzing the same animal. Two horses belonged to the Pazyryk culture (late IV—early III century BC), three—to the early Scythian period (VII century BC) and one originated from the more recent Turkic kurgan (VII century AD).

Besides, we studied DNA of a 40,000 years old ancient horse from the Denisova cave. The Denisova cave is located in the North Western part of the Altai Mountains on the right bank of the Anui River. Despite relatively little size (a central hall and two short side galleries), the cave has become a unique source of Pleistocene fossils with well-preserved DNA. The floor of the cave consists of 6 m thick sediments with clearly defined layers, dated by radiocarbon and RTL methods from modern to 280 thousand years old [17].

Using hybridization capture and high throughput sequencing we have obtained full mitochondrial genomes including control regions for six ancient domestic horses and a single wild Pleistocene horse and defined their phylogenetic position relative to previously described modern and ancient horses.

## Results

### Complete mitochondrial genome sequencing

Ancient DNA from seven samples (Table 1) was isolated according to the previously published protocol [18] with some modifications: ammonium EDTA salt was used during the bone powder dissolution stage [19], which allowed to reduce the dissolution time from 18–24 h down to 1.5–3 h under the same conditions (500 mg bone powder in 10 ml of buffer at 55°C). Mitochondrial DNA enrichment was performed using hybridization capture with modern horse mitochondrial DNA as probes [20]. Sequencing was performed on the Illumina MiSeq platform. Sequence data processing was performed in PALEOMIX (Table 2) with DNA fragmentation and misincorportation patterns analyzed with MapDamage2 (S1 File).

**Table 1:** Characteristics of samples.

Sample name	Origin	Culture	Site GPS coordinates	Age	Sample material type	Accession number (NCBI GenBank)	Accession number (NCBI Sequence Read Archive)	Mitogenome recovery, %^a^
HUk1	Ak-Alakha 1, Kurgan 1	Pazyryk	49.285356, 87.535506	IV-III centuries BC	femur bone	KT985979	SRR12116914	99.5
							SRR12116910	
							SRR12116909	
HUk2	Verkh-Kaldzhin 1, Kurgan 3	Pazyryk	49.368536, 87.552011	IV-III centuries BC	femur bone	KT985980	SRR12116908	100
							SRR12116907	
							SRR12116906	
HUk3	Ak-Alakha 2, Horse 6	Scythian	49.302661, 87.568947	VII century BC	femur bone	MK449357	SRR12116905	99.3
HUk4	Ak-Alakha 2, Horse 3	Scythian	49.302661, 87.568947	VII century BC	femur bone	MK449358	SRR12116904	98.6
HUk5	Ak-Alakha 1, Kurgan 3	Turkic	49.285356, 87.535506	VII century AD	sesamoid 1 bone	MK467453	SRR12116903	99.5
HUk6	Ak-Alakha 2, Horse 4	Scythian	49.302661, 87.568947	VII century BC	femur bone	MK467454	SRR12116913	99.8
HD2	Denisova cave, layer 11.1	wild	51.397500, 84.676111	30–50 kya	tooth	MK467455	SRR12116915	99.2
Modern^b^	Novosibirsk, Russia	-	-	present	muscle tissue	MT176385	SRR12116911	100
Modern un/en^c^	Novosibirsk, Russia	-	-	present	muscle tissue	MT176385	SRR12116912	100

^a^Based on combined sequencing results.

^b^modern horse DNA with enrichment.

^c^modern horse DNA without enrichment.

**Table 2:** Sequencing statistics.

Sample	Total read pairs	Collapsed reads	Mapped reads	% mapped	Unique hits	Coverage	Average length	% terminal deamination	% reference covered 1X
HUK1	836239	265330	2628	0.31	1175	8.28	117.40	8.81	99.59
HUK2	872737	683393	20725	2.37	8150	75.08	153.48	7.69	99.99
HUK3	492871	443296	25358	5.14	23245	83.42	59.79	10.53	98.99
HUK4	434700	403635	5635	1.30	5393	18.03	55.68	11.93	91.19
HUK5	438645	409881	22175	5.06	21228	85.78	67.32	5.00	99.80
HUK6	504377	456988	17145	3.40	15160	60.03	65.96	7.42	97.89
HD2	566163	488158	20700	3.66	16564	63.46	63.83	22.87	99.18
Modern	1215576	1164672	220697	18.16	212153	1553.94	122.03	0.42	100.00
Modern_un/en	70843	57920	2054	2.90	2035	19.06	156.01	2.03	99.09

### Library enrichment and contamination control

Enrichment efficiency was tested on modern horse DNA originating from the same individual that was used for the hybridization capture probe generation. Table 2 represents results of sequencing of both enriched (Modern) and non-enriched (Modern un/en) DNA. Without enrichment, percentage of mitochondrial DNA (2.90%) corresponded to about 9400 mitochondrial genomes per diploid nuclear genome—compared to 3650 ± 620 in human skeletal muscle [21]. The enriched library contained 18.2% of mitochondrial reads, thus enrichment efficiency was 6X. This efficiency is similar to 16% reported by the authors of the protocol [20], and overall imperfection of the enrichment can be explained by the presence of long adapters.

Since the enrichment was carried out based on the mtDNA of a modern horse, it is impossible to exclude contamination of the ancient DNA libraries with modern horse DNA. Although the ancient DNA was preliminary indexed, non-indexed contaminant sequences of modern DNA could produce chimeric products during subsequent amplification. To verify this, we analyzed the sequence of the modern horse DNA used for enrichment (Table 1) and identified 55 sites distinguishing its mitochondrial genome from the HUk1. Of the 2,113 reads covering these sites, only 36 reads contained characteristic “modern” substitutions, 25 of which also contained other substitutions belonging to at least 3 different types of mtDNA in the GenBank and differing from our modern horse. Only 11 reads (0.5%) corresponded to our sample, and these were fairly short reads that could also belong to another contaminating mtDNA. Thus, we assume that the enrichment procedure did not introduce significant contamination in the aDNA.

### Phylogenetic analysis

**Fig 2:**
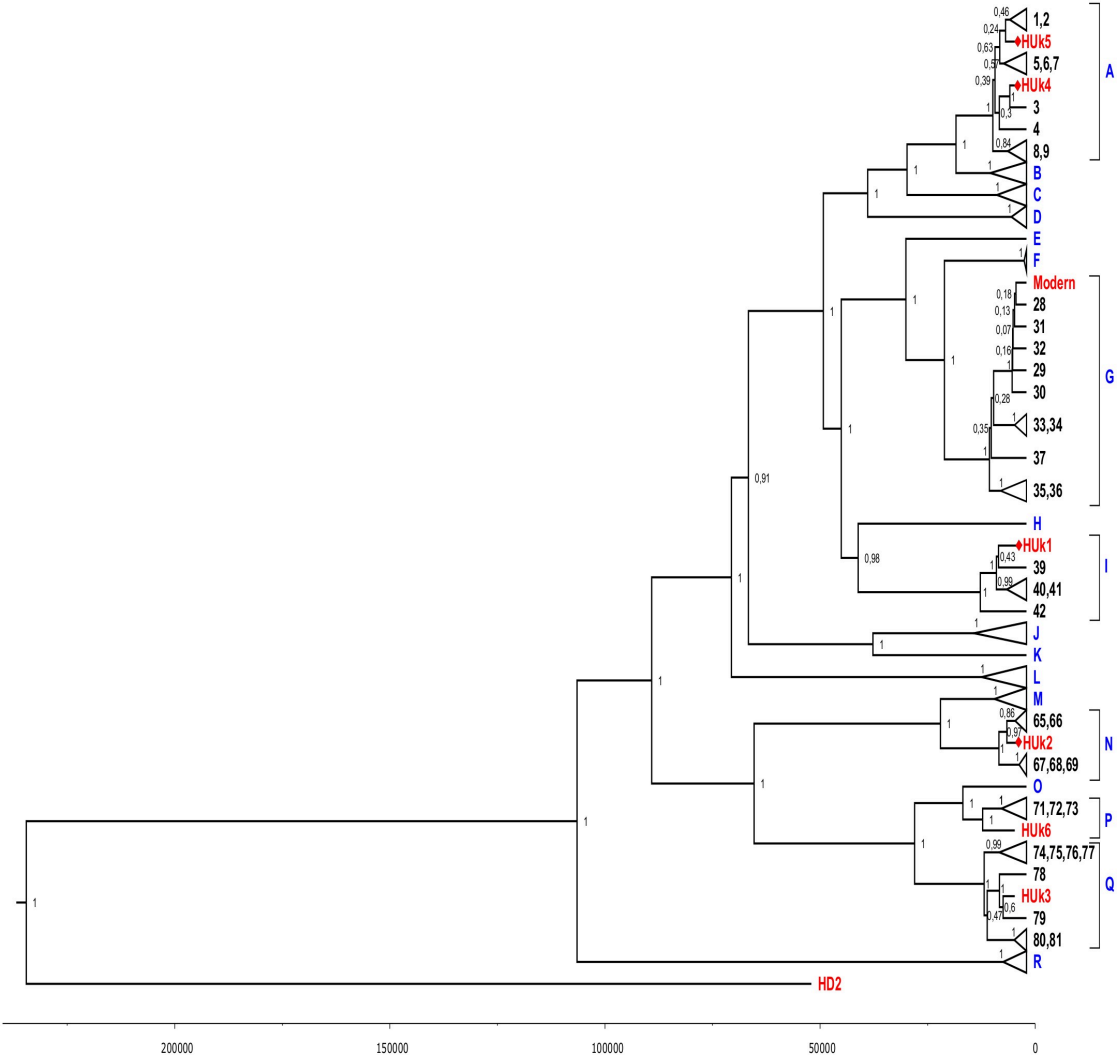
BEAST Bayesian phylogenetic tree of complete mitochondrial genomes. The tree was constructed for ancient horses studied here (red) together with published genomes of modern horses from different regions of the world [8]. In blue: mitochondrial haplogroups A-R as denoted in [8]. A published donkey (*Equus asinus*) mitochondrial genome (NC_001788.1) is used as the outgroup (not displayed). The numbers near the branch nodes of the tree indicate the posterior probability of the topology obtained by the Bayesian method. The scale at the bottom of the figure is the timeline where dates are marked in years before the present.

Using seven mitochondrial genomes of ancient horses obtained in this research and a set of genomes of modern breeds [8] we constructed a phylogenetic tree (Fig 2).

Haplogroup divergence ages for almost all haplogroups are within the last one hundred and fifty thousand years, while the wild horse haplotype HD2 separated from all other haplogroups about 255±128 thousand years ago. Phylogenetic analysis has demonstrated that the mitogenome of the wild horse HD2 does not belong to any of the modern haplogroups, but takes a basal position relative to other genomes. The specimen HD2 was found in the Denisova Cave in the layer 11.1, which was dated as around 29–40 thousand years. Obviously, such a position relative to modern horses proves its antiquity, but, at the same time, it is not the ancestral genome of any of the existing haplogroups, indicating that the lineage was lost and not present in modern populations. Paleontological data indicate that many horse maternal lineages widespread in the Late Pleistocene did not survive the dramatic climatic changes of the last glacial maximum [8].

The second surprising fact is that the Ukok samples exhibit a striking diversity: 6 horses belong to five different haplogroups—A, I, N, P, and Q.

We also determined the phylogenetic position of the modern horse, which DNA was used for the library enrichment. It belongs to the haplogroup G, different from the ancient horses, once again confirming the absence of contamination during the enrichment process.

Addition of published mitochondrial genomes used in [11] provides some additional context (S1 Fig). The wild horse from the Denisova cave formed a clade together with the 33-kya sample from the New Siberian Islands. Compared to our domestic horse samples from Altai, previously published ancient samples cover earlier dates and, not surprisingly, are more diverged from modern breeds. Overall diversity in the Ukok horses is comparable to the ancient wild samples within locations of the Ural Mountains and the New Siberian Islands, although the latter span much longer time periods.

### Phylogeography of horse haplotypes

**Fig 3:**
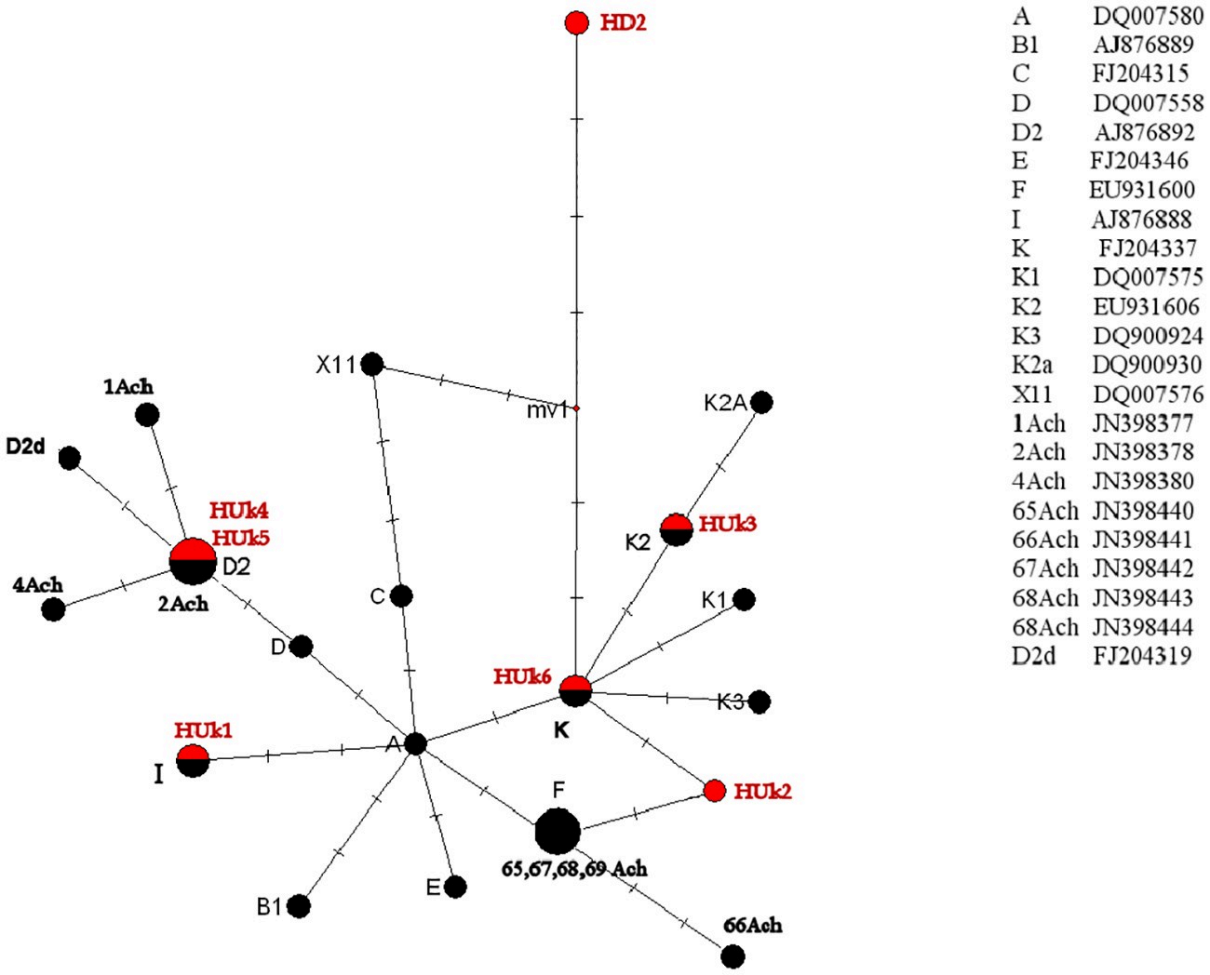
A median joining phylogenetic network of a hypervariable mtDNA region fragment. The sequences from [6] are shown on the right. The size of the circles is proportional to the number of individuals. Additional sequences are taken from the complete mitochondrial genomes [8]: 65,67,68,69 Ach—haplotypes of haplogroup N; 1,2,4 Ach—haplotypes of haplogroup A (see also Fig 2).

It should be noted that modern breeds and haplogroups of horses are so mixed around the world that it is virtually impossible to obtain any data on the primary habitat and genealogical relationships by comparison of modern lines only. Since the number of available complete mitochondrial genomes of ancient horses is quite limited, we integrated our data with fifteen sequences of 247 bp mtDNA hypervariable control region fragments from the article of Achilli and coauthors [6], who used a 247 bp fragment from the hypervariable control region of the mtDNA for 1754 modern and 207 ancient horses and identified a total of 87 haplotypes. We extracted this fragment from the mitochondrial genomes of our ancient samples together with closest relative modern samples from the article of Achilli and coauthors [6] and reconstructed the minimal median joining network that allows us to identify the possible relationships between haplotypes of our samples (Fig 3).

One of Pazyryk horses, the sample HUk2, possesses a unique haplotype first described here and it has no analogues among the ancient and modern horses. We confirmed the authenticity of the HUk2 haplotype by detailed revision of the original reads at positions 108 and 210, where mutations occurred that distinguish it from the haplotypes K and F. The position 108 is covered by 133 reads and all of them contain T, which is specific to this haplotype. The position 210 is covered by 134 reads containing C and only 4 reads with T. Thus, we believe that these mutations are real, not an artifact resulting from modification of ancient DNA. For the final determination of the origin of HUk2, we included previously published haplotypes belonging to the complete mitochondrial haplogroup N together with HUk2 (see Fig 2). Since all of those samples belong to the control region haplogroup F, we can consider HUk2 as a member of haplogroup F as well. Further in text, we will refer to HUk2 haplotype as F1.

We also see that HD2 has no analogues and is a very early diverged lineage basal to so far described domestic horses. Closest haplotypes are the X11 (late Pleistocene, absent from modern populations) and K (Iron Age sample from Tuva, Russia).

### Temporal and geographical context

Based on the summary table of ancient haplotypes [6], we found all similar haplotypes from Western and Northeastern Siberia, China, Kazakhstan, Tuva, and Altai dating from the late Pleistocene to 2300 BP, i.e. contemporary to our Pazyryk samples or more ancient. The results are shown in Table 3 and Fig 1. All mitochondrial haplotypes and haplogroups considered for comparison in the results and discussions are taken from [6].

**Table 3:** Phylogenetic position and geographical location of closely related samples.

Sample	Complete mitochondrial haplogroup^a^	Control region haplotype^b^	Geographical location	Accession	Date	The total number of animals carrying this haplotype^c^
HUk1	I	I	Yujiazhuang site, China	EU931607	500 BC	7
			Berel site, Kazakhstan	AJ876888	300 BC	
HUk2	N	F^d^	Banchneng site, China	EU931598	500 BC	9
			Banchneng site, China	EU931600	500 BC	
			Tartas1 site, West Siberia, Russia	FJ204327	2000BC	
HUk3	Q	K2	Yujiazhuang site, China	EU931608	500 BC	3
			Xiaoshuanggucheng site, China	EU931606	500 BC	
			Tartas1 site, West Siberia, Russia	FJ204322	2000BC	
HUk4, HUk5	A	D2	Siberia, Mongolia	FJ204345	400–300 BC	5
			Berel site, Kazakhstan	AJ876892	300 BC	
		D2d^d^	Denisova Cave, Altai, Russia	FJ204319	3000 BC	2
			Xindianzi site, China	EU931587	500 BC	
HUk6	P	K	Tuva, Russia	FJ20433	619–608 BC	1
HD2	**?**	X11^d^	Ulakhan-Sullar, Adycha River, Yana Basin, North-East Siberia, Russia	DQ007576	Late Pleistocene	1

^a^complete mitochondrial DNA haplogroup according to [8].

^b^control region mtDNA haplotype according to [6].

^c^the total number of animals carrying this haplotype among 207 samples of ancient horses [6].

^d^not identical, but very similar haplotypes.

As shown in the Table 3, the HUk1 sample carries haplotype I, which is also shared by individuals from Kazakhstan (Berel site) and China, who lived at the same time. In ancient times, this haplotype was more common in Europe than in Asia: a sample with haplotype I, dated 1500 BC, was found in Moldova, and four other carriers were described in horses from the Middle Ages from Moldova and Hungary [6]. Among the previously described ancient specimens, the haplotype F (HUk2) had no subfamilies and was discovered in the Copper and Iron age samples from Western Siberia (2000 BC) and in China (Inner Mongolia) [6]. In more ancient times, this haplotype was widely distributed in Europe, for example, out of seven studied haplotypes of 3600–3100 BC Ukraine horses, four belonged to F [6]. Haplotype D2 was found in the two neighboring regions—in Kazakhstan and Mongolia [6]. Besides, same haplotype was observed in much younger samples in Armenia (2 samples, 1950–1750 BC) and Spain (1 sample, 1750–1590 BC) [6]. Haplotype K (HUk6) was found at about the same time as in the Ukok in only one, fairly closely located place in Tuva (Southern Siberia) [6], and haplotype K2 (HUk3) was described in Western Siberia and China but nowhere else [6]. The table also shows the haplotype D2d (3000 BC), which was discovered in the Denisova cave and, apparently, belonged to a wild horse. This haplotype is present in 2500 years-younger Chinese samples but nowhere else [6]. The relations between the haplotypes and the locations are illustrated in Fig 1.

## Discussion

Our data indicate that from five discovered equine haplotypes from the Ukok Plateau, some control region haplotypes (I, F, and D2) were shared between concurrent and earlier populations of horses from Europe, while haplotypes K and K2 are absent from European populations and may represent specific Eastern haplotypes. No continuity was found between the early Scythian (VII century BC) and Pazyryk (IV-III century BC) horse haplotypes, while haplotype D2 was found in both Scythian and Turkic (VII century AD) kurgans, which are 1500 years apart. Limited size of our sampling prevents us from making far reaching conclusions based on these observations. It is noteworthy that the haplotype I was previously described in the Berel’ site, which also belongs to the Pazyryk culture [22] and is located just about 100 km away from the Plateau Ukok. It looks plausible that the haplotypes I, F, and possibly D2, discovered in 1500 years older horses from the burials in Spain and Armenia [6], appeared in these locations together with the nomads who brought to South Siberia ethno cultural components related to the Achaemenid culture that manifested in the Pazyryk material culture—ornamental traditions and imported goods, for example carpets, textile, golden and silver products [23]. On the other hand, it is possible that D2 might have migrated to Altai from Mongolia. Alternatively, it can represent an independent local haplotype.

As shown on the map (Fig 1), the haplotypes K and K2 were also found in ancient Tuva and Western Siberia (Tartas site, Novosibirsk region) [6]. As haplotype K has not been described anywhere else so far, it might represent an autochthonous maternal lineage involved in domestication process. The haplotype K2 was found in two regions of China along with haplotypes I and F. This similarity in haplotype distribution can be explained by known acquisition of horses by China from nomads. In ancient times, Chinese could not grow good horses, and since the IV century BC cavalry played a growing role in the Chinese army, hence nomad horses became key trade objects [24].

It should be also noted that the haplotype of the wild Pleistocene horse D2d (300 BC, Denisova Cave, Altai) was also discovered only in China (Inner Mongolia, 500 years BC) but not in other places [6]. This haplotype differs from D2 by a single C-to-T transition, and thus the autochthonous haplotype D2 might have been ancestral to D2d.

The horses from Pazyryk burials are quite interesting for domestication history. For Pazyryk people, horse was not only a means for transportation and nutrition. Importance of this animal for culture bearers in day-to-day life and at war determined its high status in the society. It is not a coincidence that horse had an uttermost role in burial, accompanying the deceased owner to another world. It was demonstrated [25, 26] that well-preserved horse skeletons can be split into four groups based on morphology. Two groups are clearly riding horses, characterized by a narrow forehead and high growth (1.40–1.55 m). These horses do not have analogs among the local (Altai, Kazakh, Mongolian) breeds, they obviously belonged to notables or chiefs, and were held in good conditions. Looking at the representative series of horses of the Pazyryk culture from the Ukok Plateau and Southern Altai [25, 27, 28], paleozoologists suggested that both Western and local origin are possible for the “tall” horses. Skeletons of groups 3 and 4 are more numerous, these horses are squat (1.28–1.36 m), with a short neck—possibly carriage horses. According to the hoof morphology, these were freely grazed, often left to fend for themselves and thus experienced multiple starvations. Considering extremely harsh climatic conditions of Altai, only well adapted mares could bear a foal, i.e. those whose maternal lineages were derived from local populations. Unfortunately, the morphological description of the bones used in present work is missing, but we can assume that haplotypes I and F belong to riding horses from Europe, while haplotypes K and K2 represent local maternal lineages. Further analysis of a large amount of morphologically described samples from the Ukok plateau burials and comparison to wild Altai horses from Holocene looks very promising for resolving this issue.

## Methods

### Information about samples

All studied bone and tooth samples of ancient horses (HD2, HUK1, HUK2, HUK3, HUK4, HUK5, HUK6) are stored in a repository located in the Russian Federation, Novosibirsk 630090, Akademgorodok, Academician Lavrentiev Avenue 17, Institute of Archeology and Ethnography of the Siberian Branch of the Russian Academy of Sciences. All these samples are the property of the Institute and are available for viewing. The study of these samples was carried out at the Institute of Molecular and Cellular Biology SB RAS (Russian Federation, Novosibirsk 630090, Akademgorodok, Acad. Lavrentiev Ave. 8/2). Both institutes belong to the same academic structure and are located on the territory of the Russian Federation. The excavation leaders (Natalia V. Polosmak, Vyacheslav I. Molodin, Sergei K. Vasiliev, Michael V. Shunkov) are co-authors of this article and have given their consent to conduct a joint interdisciplinary study, on the territory of the Russian Federation this is sufficient to carry out a joint project. No permits were required for the described study, which complied with all relevant regulations. The person who provided the samples and is responsible for the sample collection is the co-author of this article, Doctor of Historical Sciences, Head of the Paleometal Archeology Department of the Institute of Archeology and Ethnography SB RAS, Vyacheslav Ivanovich Molodin. A modern sample (Modern) was bought on the market as horse meat product. Information about the samples and the sampling sites are given in the Table 1.

### Ethics statement

All experiments were approved by the Committee on the Ethics of Animal Experiments of the Institute of Molecular and Cellular Biology (IMCB) SB RAS, Russia.

### DNA extraction and sequencing library preparation

DNA of modern horse was extracted using DNeasy^®^ Blood & Tissue Kit (Qiagen, Netherlands) according to the manufacturer’s guidelines.

For ancient DNA (aDNA) extraction, the surface layer of the bone was removed with diamond blade, compact substance (~1 cm^3^) was cut out and ground to powder in a stainless steel mortar. 10 ml of solution containing 0.5 M ammonium EDTA, 0.5% sodium lauroyl sarcosinate, and 0.5 mg/ml proteinase K was added to 0.5 g of the bone. The resulting suspension was incubated and stirred for 2–2.5 hours at 55°C until complete bone dissolution. After the solution became clear, the samples were concentrated in centrifugal filter units Amicon Ultra (15 ml, 3 kD cutoff; Millipore, Billerica, MA), and purified with MinElute PCR Purification Kit (Qiagen, Venlo, the Netherlands).

Sequencing libraries were constructed with TruSeq^®^ Nano DNA Sample Preparation Kit (Illumina, San Diego, USA) according to the manufacturer’s guidelines with a few changes: samples were purified via MinElute PCR Purification Kit (Qiagen, Germany) instead of purification with the AMPure XP beads (Illumina, San Diego, USA), 12 cycles of library amplification were performed, initial DNA fragmentation and library size selection steps were skipped.

### Immobilization of modern horse mtDNA on magnetic beads

Prior to sequencing, library enrichment was performed by hybridization with biotinylated mitochondrial DNA of the modern horse immobilized on Dynabeads^®^ Streptavidin magnetic beads (Thermo Fisher Scientific, Waltham, MA) according to [20].

For probe preparation, horse mitochondrial DNA was PCR-amplified as four fragments (about 4 kbp each) in PCR with the following primers: 1F-gaggagcctgttccataatcg, 1R-ggttagggggaggagtagg, 2F-tcccatccacaaacaacataaa, 2R-gaggcttggagaagggtgaag, 3F-tgaccacccacaggtatcca, 3R-acgttggtggagtgttctagtt, 4F-ggcagcatttttgccggatt, 4R-gatggtggggtttatcgggg. PCR solution included 10–20 ng DNA, 1 μM primers and Kapa Ready Mix (KAPABIOSYSTEMS). Cycling conditions: 3 min at 95°C, then 35 cycles of: 20 sec at 95°C, 30 sec at 60°C, 5 min at 72°C. An equimolar mixture of the fragments was biotinylated with Nick Translation BioNick Labeling System Kit (Thermo Fisher Scientific, Waltham, MA), purified with MinElute kit, denatured for 5 min at 95°C and immobilized on the pre-washed (3 times 2×SSC) magnetic beads in 2×SSC solution for 30 min at room temperature. Optical density was measured in the initial solution and after three washes (1 ml 2×SSC each), and the amount of the immobilized DNA was estimated as 1.2–1.7 μg in 50 μl of the initial beads suspension.

### Hybridization

Indexed libraries with 900–1500 ng DNA in 30 μl of 10 mM Tris-HCl (pH 8.5) were denatured for 5 min at 95°C and immediately cooled on ice. After that, 10 μl of magnetic bead suspension with 0.24–0.34 μg biotinylated mtDNA were added; 0.1 volume of 20×SSC solution was then added; and hybridization was performed for 48–72 h at 65°C. Then, magnetic beads were washed with 3×100 μl of 2×SSC at 65°C, 2х100 μl of 0.2×SSC at room temperature and eluted in 30 μl of water for 5 min at 96°C. Amplification of the enriched aDNA fragments from the sequencing library was performed in 50 μl final volume with Phusion HF Buffer, 0.25 mM 4dNTP each, 1 μM primers (complementary to Illumina adapters), 1 U Phusion DNA polymerase for 16 cycles (pre-cycle 30 sec at 98°C; cycle conditions: 15 sec at 98°C, 20 sec at 67°C, 1 min at 72°C).

### Sequencing

Sequencing libraries were quantified with RT-PCR and sequenced on Illumina MiSeq (Reagent Kit v2, 500-cycles) according to the manufacturer’s protocols.

### Sequence data analysis

Initial data processing was performed with PALEOMIX pipeline v1.2.13.2 [29]: first, Illumina adapters were removed and overlapping paired reads were collapsed with AdapterRemoval v2.1.7 [30]. Next, only collapsed reads were aligned to reference horse mitochondrial genome (GenBank: NC_001640) with bwa mem v. 0.7.17-r1188 (arXiv:1303.3997 [q-bio.GN]). PCR duplicates were removed by custom script in PALEOMIX that accounts only for matching read end coordinates in reference genome. Sequence quality was recalibrated according to the deamination profile obtained with mapDamage v2.0.8 [31]. Indel realignment was performed with GATK v3.8.0 IndelRealigner [32].

Consensus sequences of mitogenomes were obtained in Geneious 8 (http://www.geneious.com/) based on 75% identity consensus with manual inspection of potential deamination sites and characteristic Illumina errors in the homopolymer tracts.

### Phylogenetic analysis

To construct phylogenetic trees of complete mitochondrial genomes, the consensus sequences were aligned together with the contemporary samples previously used to delimit mitochondrial haplogroups [8] using MAFFT v7.407 [33]. For five functional groups of sequences optimal substitution models were identified with PartitionFinder v2.1.1 [34]: HKY+I for second codons; HKY+G+I for RNA genes, first and third codons; GTR+G+I for hypervariable regions. Using these models, BEAST Bayesian tree shown in Fig 2 was inferred using an advanced software platform for Bayesian evolutionary analysis BEAST v1.10.4 [35] (10 million generations, sampling frequency—1000, first 10% trees discarded as burn-in), clock model—strict clock, tree prior—coalescent: constant size [36, 37], tree model—random starting tree. The BEAST control file was generated using the program BEAUti v1.10.4., the BEAST output was analyzed using the program Tracer v1.7.1 [38]. In Tracer, effective sample sizes (ESSs) for almost all the traces are more than 400, except traces hypervariable.gtr.rates.rateAG, hypervariable.gtr.rates.rateCT, for which ESSs are 100–200, the posterior probability density is bell-shaped, which suggest that the parameter estimates have reached the convergence. To summarize the information contained within our sampled trees we used the tool TreeAnnotator v1.10.4 [39]. The annotated tree was visualized using the program FigTree v1.4.4. ("http://tree.bio.ed.ac.uk/software/figtree/).

247 bp fragment of mitochondrial hypervariable control region was isolated based on the alignment with reference horse mitochondrial genome (GenBank: X79547, positions 15495–15741 bp). Previously identified hypervariable positions 15585, 15597, 15650, and 15604 [6] were removed. Phylogenetic network was constructed using a median joining method [40] implemented in Network v4.612 (http://www.fluxus-engineering.com/sharenet.htm).

## Supporting information

S1 Fig Phylogenetic tree with published mitogenomes. (PDF)

S1 File Mapdamage diagrams. (PDF)
