# Supplementary figures and images for: High genetic diversity of ancient horses from the Ukok Plateau
Source: PLoS One. 2020 Nov 12;15(11):e0241997. doi: 10.1371/journal.pone.0241997 (PMC7660532; doi:10.1371/journal.pone.0241997)

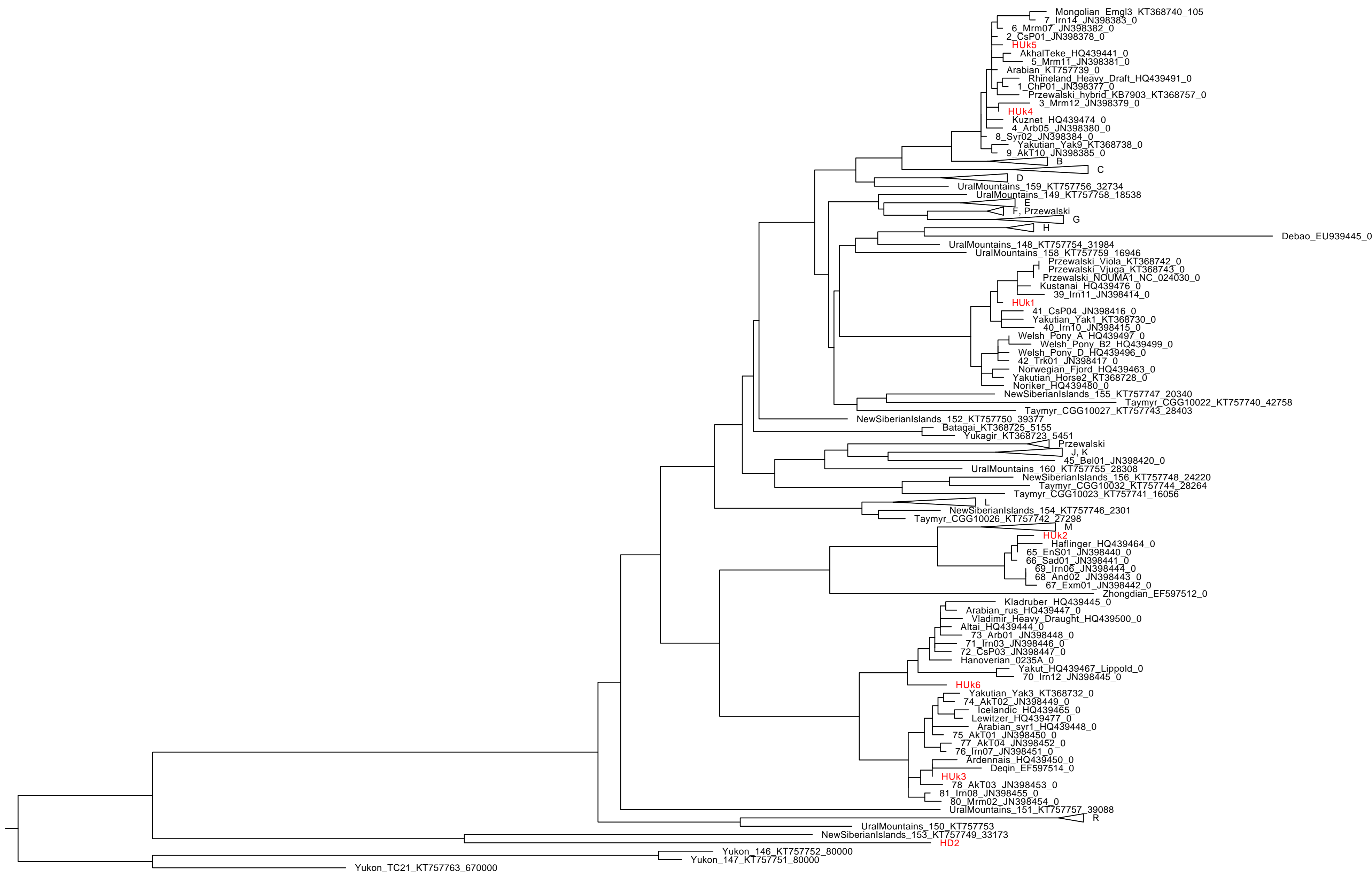

0.002

Supplement: S1 Fig — (PDF) [file pone.0241997.s001.pdf]
