# Supplementary material for: High genetic diversity of ancient horses from the Ukok Plateau
Source: PLoS One. 2020 Nov 12;15(11):e0241997. doi: 10.1371/journal.pone.0241997 (PMC7660532; doi:10.1371/journal.pone.0241997)
Supplement: S1 File — (PDF) [file pone.0241997.s002.pdf]

mapDamage plot for library 'HUK1'

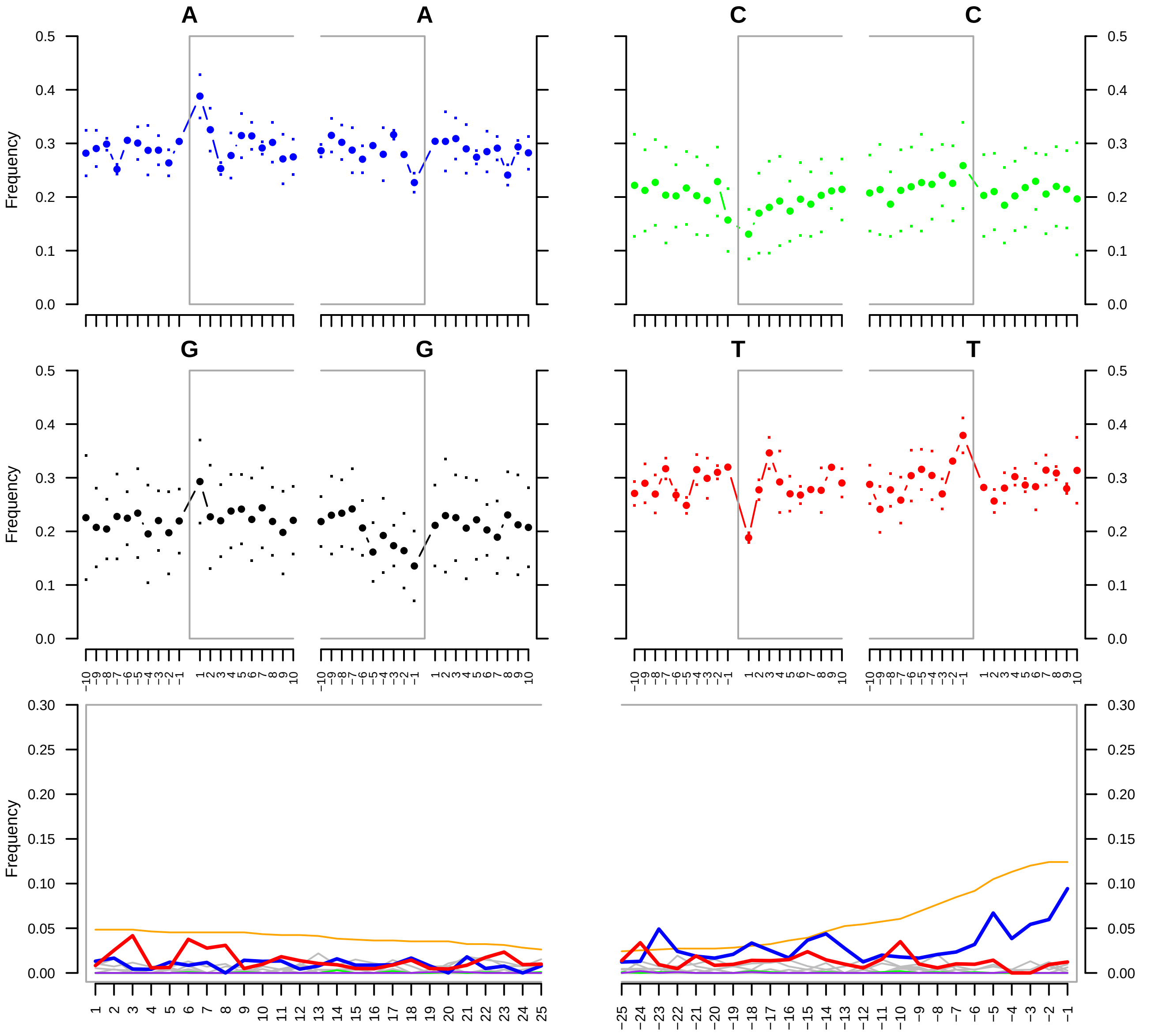

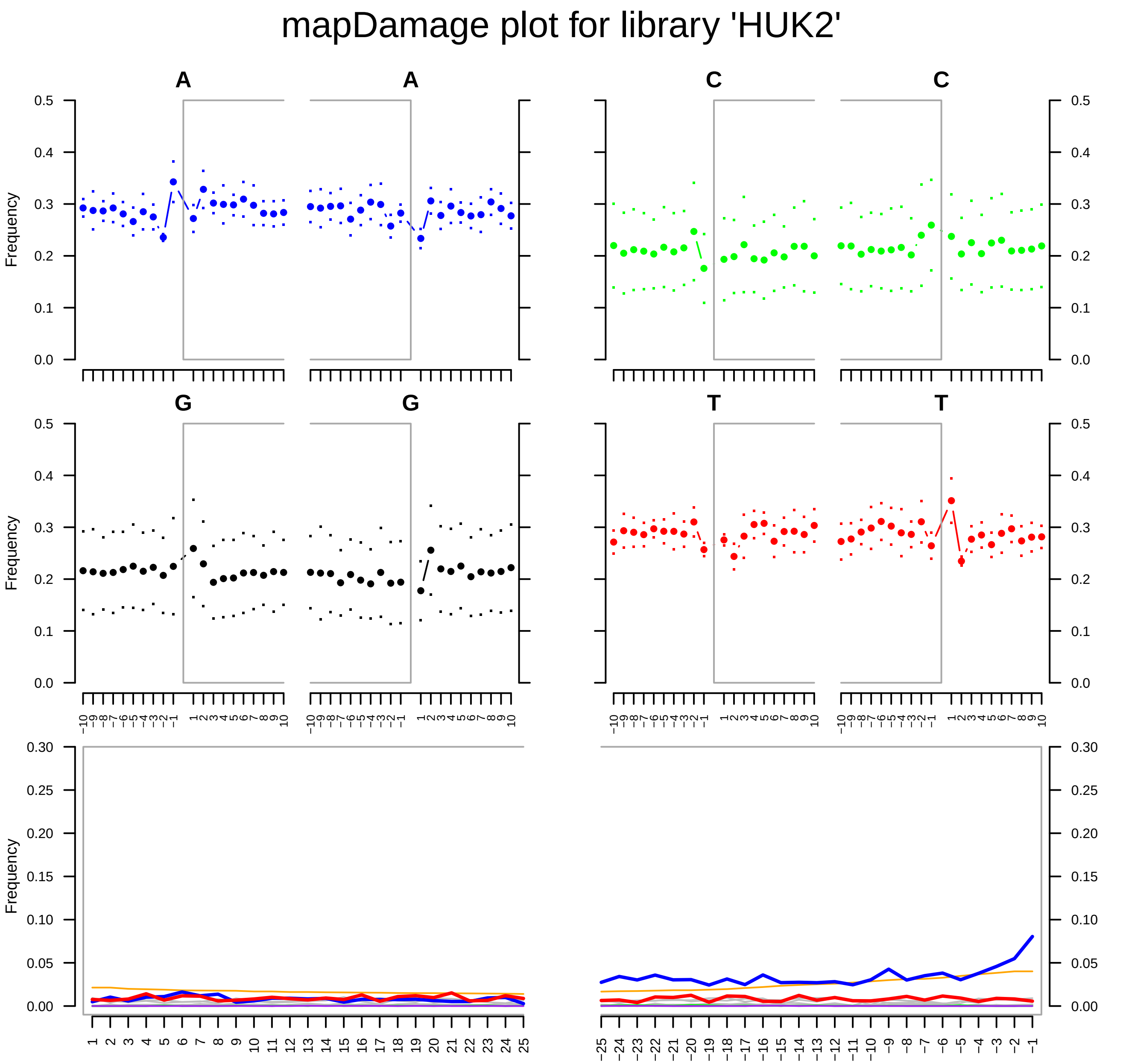
mapDamage plot for library 'HUK2'
A
A
C
C
G
G
T
T
Frequency
Frequency
Frequency
0.0
0.1
0.2
0.3
0.4
0.5
0.00
0.05
0.10
0.15
0.20
0.25
0.30

mapDamage plot for library 'HUK3'

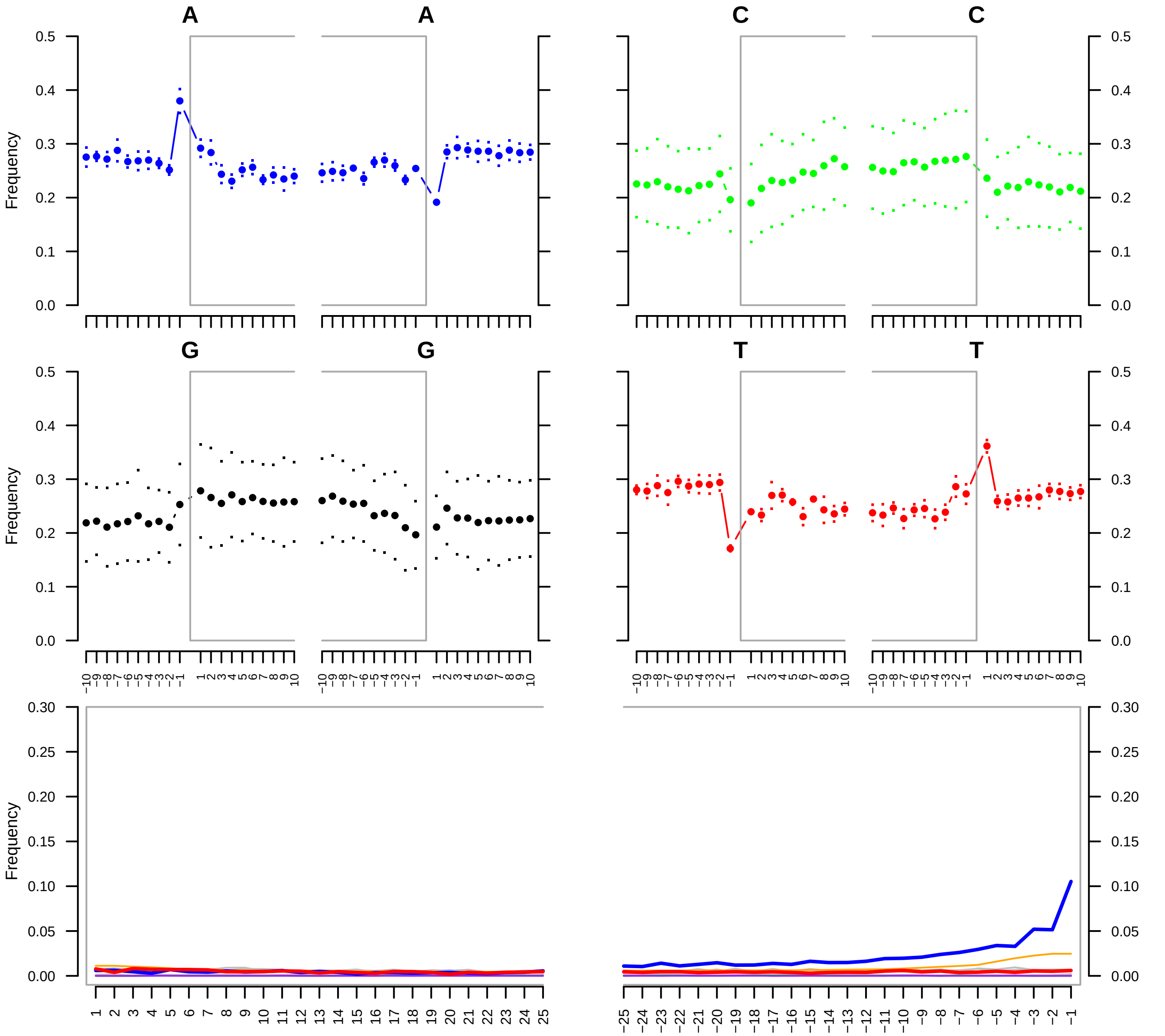

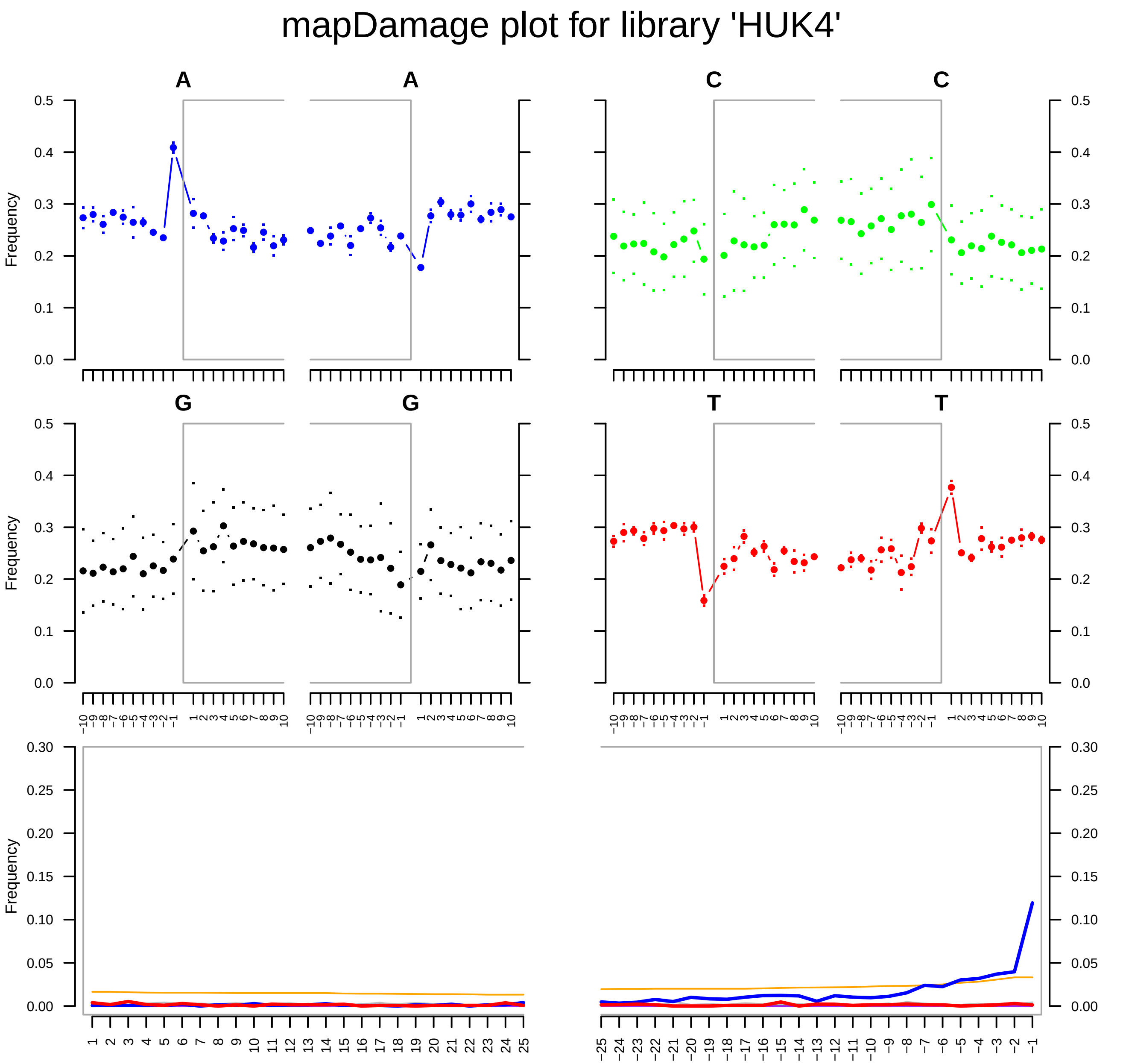

mapDamage plot for library 'HUK4'
A
A
C
C
G
G
T
T
Frequency
0.0
0.1
0.2
0.3
0.4
0.5
0.00
0.05
0.10
0.15
0.20
0.25
0.30

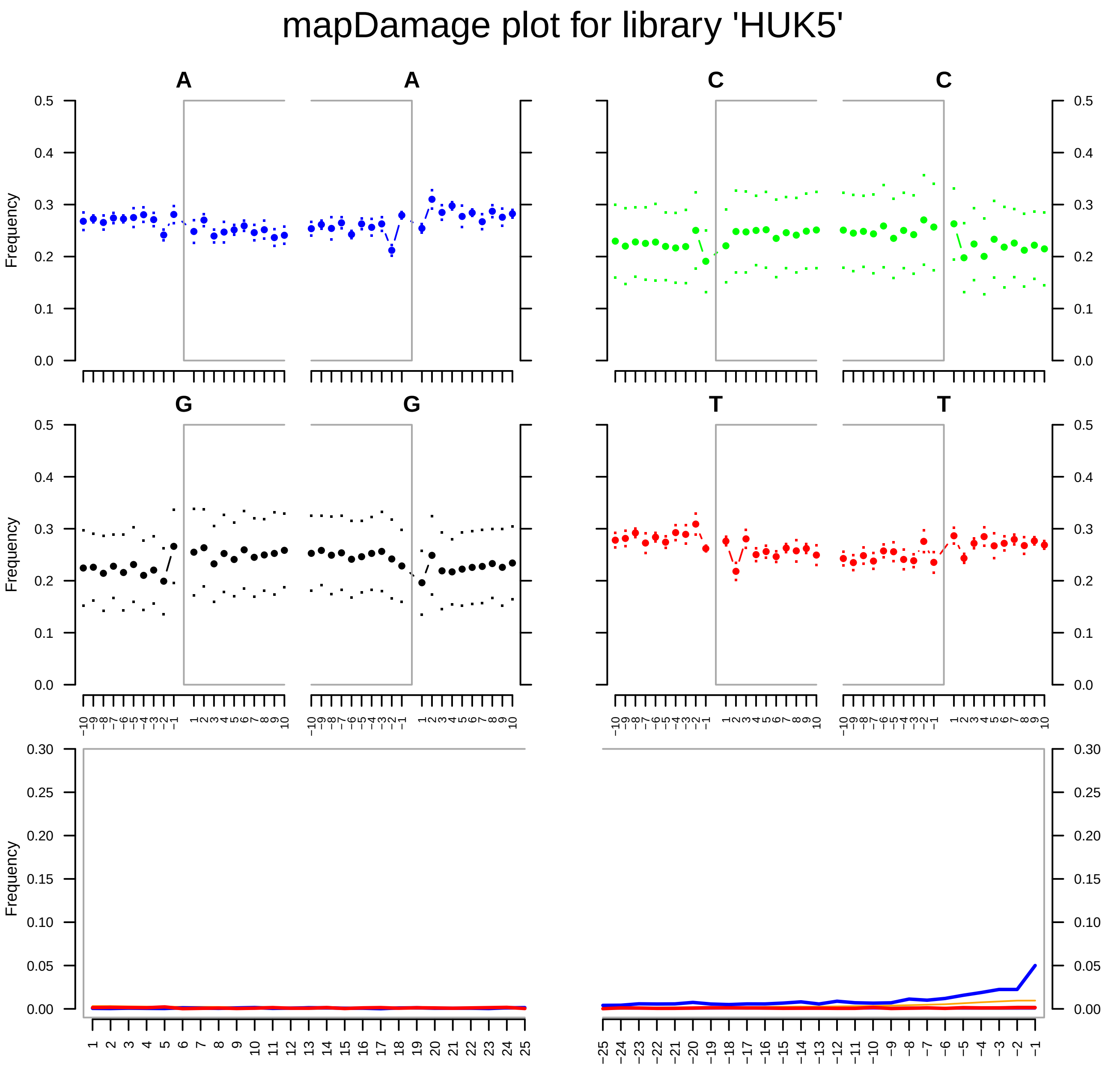

mapDamage plot for library 'HUK5'
A
A
C
C
G
G
T
T
Frequency
0.0
0.1
0.2
0.3
0.4
0.5
0.00
0.05
0.10
0.15
0.20
0.25
0.30

mapDamage plot for library 'HUK6'

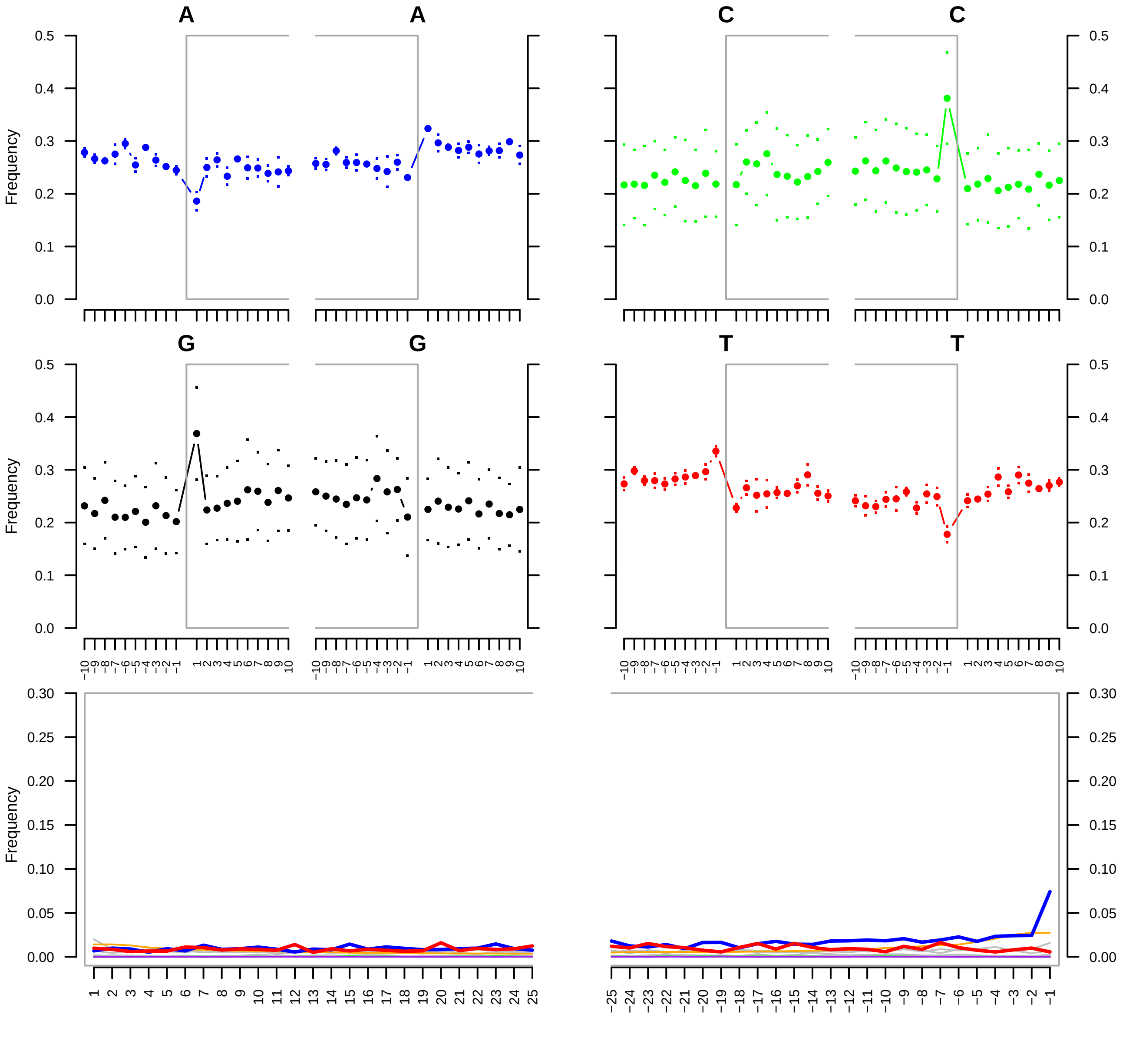

mapDamage plot for library 'HD2'

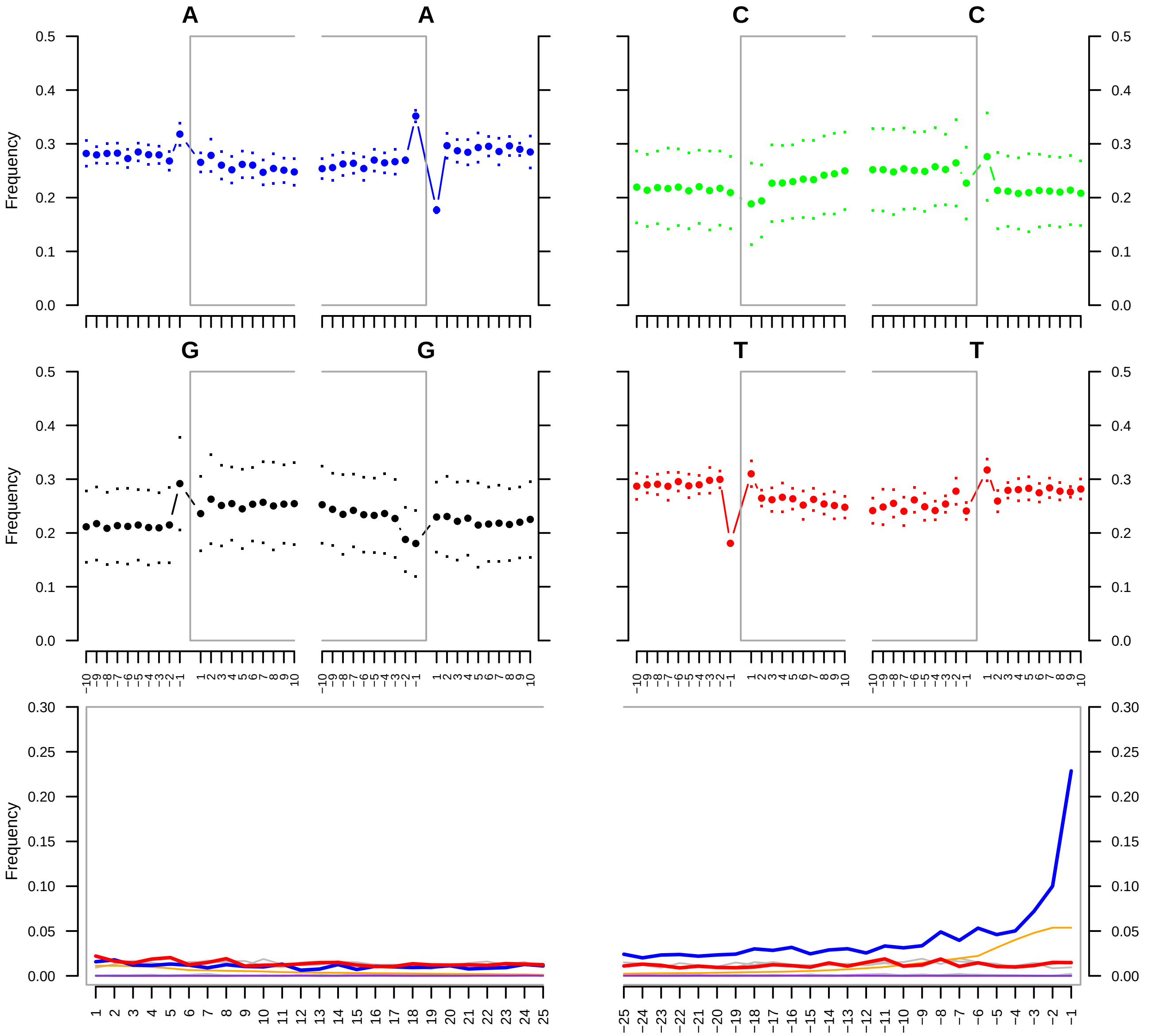

mapDamage plot for library 'modern'

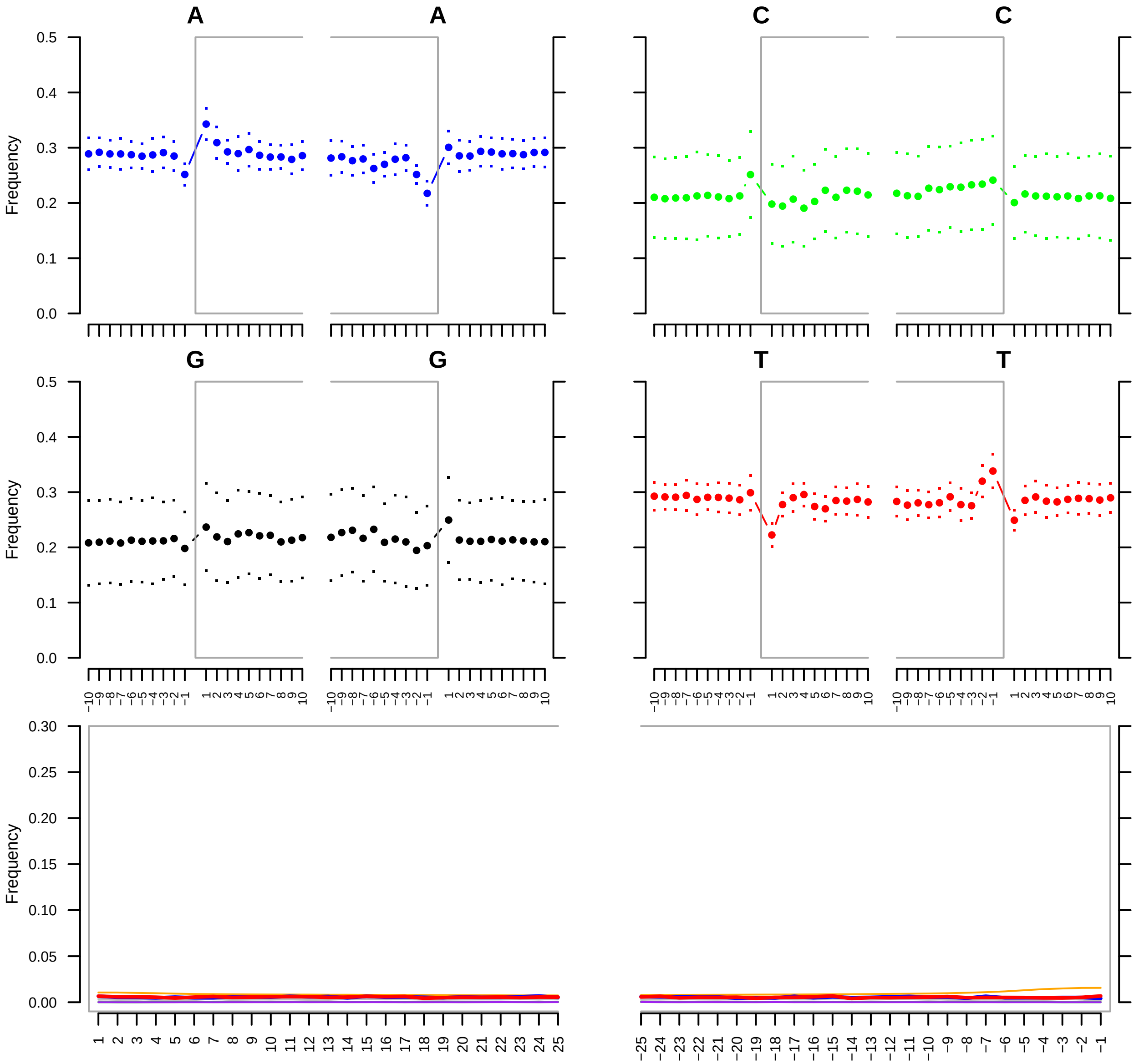

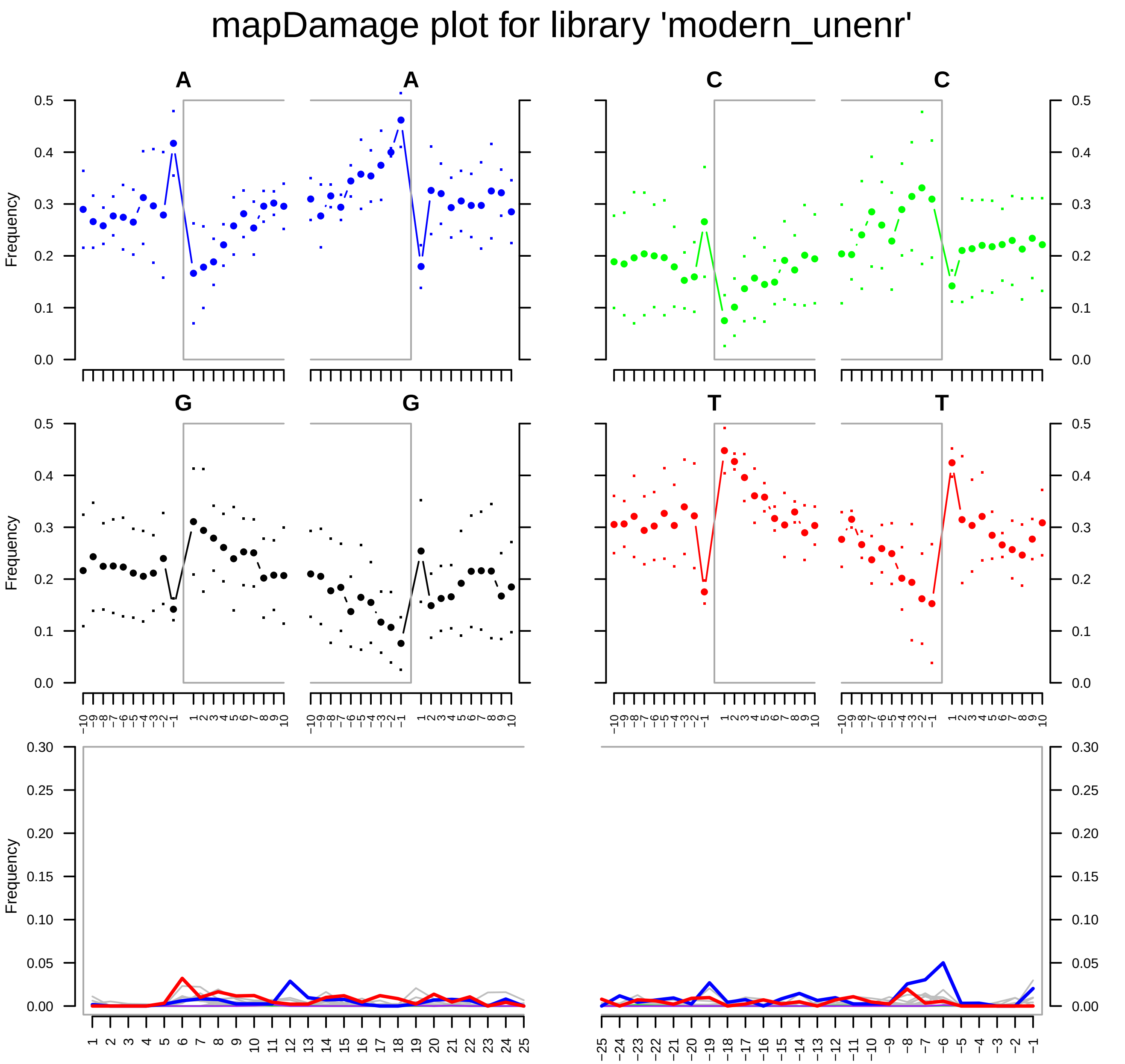
mapDamage plot for library 'modern_unenr'
A
A
C
C
G
G
T
T
Frequency
Frequency
Frequency
0.0
0.1
0.2
0.3
0.4
0.5
0.00
0.05
0.10
0.15
0.20
0.25
0.30
−10 −9 −8 −7 −6 −5 −4 −3 −2 −1 1 2 3 4 5 6 7 8 9 10
1 2 3 4 5 6 7 8 9 10 11 12 13 14 15 16 17 18 19 20 21 22 23 24 25
−25 −24 −23 −22 −21 −20 −19 −18 −17 −16 −15 −14 −13 −12 −11 −10 −9 −8 −7 −6 −5 −4 −3 −2 −1
